# NMDA receptor GluN2A/GluN2B subunit ratio as synaptic trait of levodopa-induced dyskinesias: from experimental models to patients

**DOI:** 10.3389/fncel.2015.00245

**Published:** 2015-07-06

**Authors:** Manuela Mellone, Jennifer Stanic, Ledia F. Hernandez, Elena Iglesias, Elisa Zianni, Annalisa Longhi, Annick Prigent, Barbara Picconi, Paolo Calabresi, Etienne C. Hirsch, Jose A. Obeso, Monica Di Luca, Fabrizio Gardoni

**Affiliations:** ^1^Dipartimento di Scienze Farmacologiche e Biomolecolari (DiSFeB), Università degli Studi di MilanoMilano, Italy; ^2^Movement Disorders Group, Neurosciences Division, Center for Applied Medical Research (CIMA), Center for Networked Biomedical Research on Neurodegenerative Diseases (CIBERNED), University of NavarraPamplona, Spain; ^3^Inserm, U 1127Paris, France; ^4^CNRS, UMR 7225Paris, France; ^5^Sorbonne Universités, UPMC Univ Paris 06, UMR S 1127Paris, France; ^6^Institut du Cerveau et de la Moelle Épinière, ICMParis, France; ^7^Fondazione Santa Lucia, IRCCSRome, Italy; ^8^Clinica Neurologica, Dipartimento di Medicina, Università degli Studi di Perugia, Ospedale Santa Maria della Misericordia, Localitá, Sant’Andrea delle FrattePerugia, Italy

**Keywords:** NMDA receptor, Parkinson’s disease, levodopa-induced dyskinesias, 6-OHDA rat model, MPTP monkey model, patients, cell-permeable peptides, striatum

## Abstract

Levodopa-induced dyskinesias (LIDs) are major complications in the pharmacological management of Parkinson’s disease (PD). Abnormal glutamatergic transmission in the striatum is considered a key factor in the development of LIDs. This work aims at: (i) characterizing N-methyl-D-aspartate (NMDA) receptor GluN2A/GluN2B subunit ratio as a common synaptic trait in rat and primate models of LIDs as well as in dyskinetic PD patients; and (ii) validating the potential therapeutic effect of a cell-permeable peptide (CPP) interfering with GluN2A synaptic localization on the dyskinetic behavior of these experimental models of LIDs. Here we demonstrate an altered ratio of synaptic GluN2A/GluN2B-containing NMDA receptors in the striatum of levodopa-treated dyskinetic rats and monkeys as well as in post-mortem tissue from dyskinetic PD patients. The modulation of synaptic NMDA receptor composition by a cell-permeable peptide interfering with GluN2A subunit interaction with the scaffolding protein postsynaptic density protein 95 (PSD-95) leads to a reduction in the dyskinetic motor behavior in the two animal models of LIDs. Our results indicate that targeting synaptic NMDA receptor subunit composition may represent an intriguing therapeutic approach aimed at ameliorating levodopa motor side effects.

## Introduction

Glutamatergic transmission is greatly involved in the pathophysiology of Parkinson’s disease (PD) and more specifically in levodopa (L-DOPA)-induced dyskinesias (LIDs; Calabresi et al., 2010). After chronic treatment with L-DOPA, the glutamatergic signaling from the cortex to the striatum undergoes adaptive changes which result in the aberrant functioning of N-methyl-D-aspartate receptors (NMDARs) at the dendritic spine of striatal medium spiny neurons (MSNs; Sgambato-Faure and Cenci, 2012; Mellone and Gardoni, 2013). NMDAR antagonists have been shown to exert a beneficial effect in reducing the development of dyskinesias in experimental models of LIDs (Hadj Tahar et al., 2004; Wessell et al., 2004). Most importantly, they have been tested in clinical trials to reduce the receptor activity in L-DOPA-treated dyskinetic patients (Nutt et al., 2008). In particular, the NMDAR antagonist amantadine exerts an anti-dyskinetic effect in PD patients (Luginger et al., 2000; da Silva-Júnior et al., 2005; Sawada et al., 2010; Elahi et al., 2012).

Besides NMDAR overactivation, a large number of studies have indicated that the expression and the synaptic distribution of specific NMDAR subtypes have a role in PD and LIDs (Picconi et al., 2004; Hallett et al., 2005; Gardoni et al., 2006; Feng et al., 2014; Zhang et al., 2014). In the 6-hydroxydopamine (6-OHDA) rat model of PD, dopamine denervation influences NMDAR subunit composition depending on the extent of DA depletion (Gardoni et al., 2006; Paillé et al., 2010). Chronic L-DOPA treatment also results in an abnormal NMDAR composition at MSN dendritic spines. Specifically, GluN2B subunit is aberrantly distributed to the extrasynaptic membrane in the striatum of L-DOPA-treated dyskinetic rats, while the synaptic levels of GluN2A are augmented (Gardoni et al., 2006). Notably, alterations in the synaptic NMDAR GluN2A/GluN2B subunit ratio in the striatum correlate with the motor behavior abnormalities observed in rat models of PD and LIDs (Gardoni et al., 2006, 2012; Paillé et al., 2010). In particular, we previously demonstrated that 6-OHDA lesioned rats chronically treated with L-DOPA but not showing LIDs have a normal GluN2A/GluN2B ratio at synapses (Gardoni et al., 2006). Prevention of the aberrant NMDAR GluN2A/GluN2B subunit ratio at striatal excitatory synapses by concomitant chronic administration of L-DOPA and TAT2A, a cell-permeable peptide (CPP) interfering with the interaction between GluN2A and the scaffolding protein postsynaptic density protein 95 (PSD-95), is sufficient to significantly reduce the onset of LIDs in parkinsonian rats (Gardoni et al., 2012). Such CPP approach appears as a promising putative anti-dyskinetic treatment in PD. However, these encouraging results need validation in primate models of LIDs as well as target confirmation in post-mortem tissue from L-DOPA-treated dyskinetic PD patients. Furthermore, future clinical evaluation of the efficacy of CPPs targeting NMDARs will likely be focused on PD patients already showing a dyskinetic behavior. Accordingly, in this work: (i) we evaluated NMDAR subunit composition at striatal synapses of parkinsonian and dyskinetic rats, monkeys and PD patients; and (ii) we explored the potential therapeutic effect of TAT2A administration to rats and monkeys with established and consolidated dyskinesia.

## Materials and Methods

### Animals

Adult male Sprague Dawley rats (125–175 g; Charles River Laboratories, Calco, Italy) were used in this study. Rats were maintained on a 12 h light/dark cycle in a temperature-controlled room (22°C) with free access to food and water. All procedures were performed in accordance with the current European Law and were approved by the Italian Ministry of Health (as indicated in Dlgs N. 295/2012-A). 25 male non-human primates (Macaca fascicularis) weighing 3–6 kg and at the age of 3–7 years old (young adults) were sourced from R.C. Hartelust BV (Tilburg, Netherlands). Animals were housed in an animal room under standard conditions and treated in accordance with the European and Spanish guidelines (86/609/EEC and 2003/65/EC European Council Directives; and the Spanish Government). The Bioethics Committee of the Universidad de Navarra approved the experiments.

### Cell-Permeable Peptides (CPPs)

Active (TAT2A; YGRKKRRQRRR-KMPSIESDV) and control (TAT; YGRKKRRQRRR) CPPs were synthetized by Bachem (Bubendorf, Switzerland) according to our designed sequences. Lyophilized CPPs were solubilized in sterile deionized water to a stock concentration of 1 mM and stored at −20°C before use.

### Antibodies

The following unconjugated primary antibodies were used: mouse monoclonal (mAb) anti-GluN2B and anti-PSD-95 (1:2000, #75–097 clone 59/20 and #75028, Neuromab, Davis, CA, USA); rabbit polyclonal (rAb) anti-GluN2A (1:1000) and mAb anti-alpha-tubulin (1:10,000, #M264 and #T9026 respectively, Sigma-Aldrich, St. Louis, MO, USA); rAb anti-MAP2 (1:500, #AB5622, Merck-Millipore, Billerica, MA, USA); mAb anti-HIV1 TAT antibody (1:200, #63957, Abcam, Cambridge, UK). Goat AlexaFluor 488- and 555-conjugated anti-mouse and anti-rabbit secondary antibodies (both 1:250, #A11029 and #A21429, Life Technologies, Monza, Italy) were used for immunofluorescence studies, while goat anti-mouse or anti-rabbit horseradish peroxidase (HRP)-conjugated antibodies were bought from Bio-Rad (both 1:10,000, #172–1011 and #172–1019, Hercules, CA, USA) for western blot (WB) analysis.

### 6-Hydroxydopamine (6-OHDA) Rat Model

Rats (*n* = 100) were unilaterally lesioned with 6-OHDA (Sigma-Aldrich; 12 μg/4 μl, rate of injection 0.38 μl/min; stereotaxic injection in the medial forebrain bundle, MFB, AP: −4.4, L: +1.2; DV: −7.5) as previously reported (Picconi et al., 2008). Fifteen days after the lesion, the rats were tested with 0.05 mg/kg subcutaneous injection of apomorphine (Sigma-Aldrich), and the contralateral turns were counted for 40 min. Only those animals able to perform at least 200 contralateral turns following apomorphine injection were used for the behavioral and molecular experiments (fully lesioned rats, approximately 70% of the lesioned rats; Paillé et al., 2010). The severity of the lesion was also quantified evaluating the levels of striatal tyrosine hydroxylase (TH; #AB152, Merck-Millipore) by WB analysis.

### L-DOPA-Induced Dyskinesias and TAT2A Treatment in 6-OHDA-Lesioned Rats

Two months after the stereotaxic injection of 6-OHDA, eight fully lesioned rats were sacrificed and the ispilateral (6-OHDA I) and controlateral (6-OHDA C) striata were collected for molecular studies. The remaining fully lesioned rats were treated with 6 mg/kg L-DOPA (Sigma-Aldrich) combined with 6 mg/kg benserazide (Sigma-Aldrich), 1 s.c. injection/day for 14 days. L-DOPA-induced abnormal involuntary movements (AIMs) were evaluated on days 4, 7, 10 and 14 of L-DOPA administration using a highly validated rat AIMs scale (Cenci et al., 1998; Lundblad et al., 2002; Picconi et al., 2003; Gardoni et al., 2006). Briefly, rats were observed individually for 1 min every 20 min from 20 to 140 min after the L-DOPA injection. At each observation time point the AIMs were classified into three subtypes: (i) axial (dystonic or choreiform torsion of the upper part of the body toward the side contralateral to the lesion); (ii) limb (jerky and/or dystonic movements of the forelimb contralateral to the lesion); and (iii) orolingual (empty jaw movements and tongue protrusion). Each of these subtypes was scored on a severity scale from 0 to 4, where 0 = absent, 1 = present during less than half of the observation time (<30 s), 2 = present for more than half of the observation time (>30 s), 3 = present all the time (=1 min) but suppressible by external stimuli, and 4 = present all the time and not suppressible by external stimuli. The total AIM score for each test session was obtained by summing the scores of all observation time points. The rats that reached an AIMs score per session that was equal to or higher than 25 were included in the dyskinetic group (approximately 60% of the rats which were treated with L-DOPA). For the molecular studies, eight rats per group were sacrificed 1 h after the last daily L-DOPA injection (Gardoni et al., 2006).

Following chronic administration of L-DOPA, dyskinetic rats underwent a single stereotaxic injection of 1 nmol (*n* = 4), 5 nmol (*n* = 5), 10 nmol (*n* = 6) TAT2A or 5 nmol TAT as control (*n* = 6) in the striatum ipsilateral to the 6-OHDA lesion site (rate of injection 0.5 μl/min; AP = +0.2, *L* = +3.5, DV = −5.7) at day 15–19 of L-DOPA treatment. Six untreated dyskinetic rats were used as further control. L-DOPA administration was continued for 1 day after CPP injection (30 h). To evaluate the effects of these CPPs on LIDs, behavioral assessments (AIM score) on TAT/TAT2A-injected rats were carried out in double-blinded conditions the day before the surgery (18 h before CPPs stereotaxic injection), on the day of the surgery and the following day (6 and 30 h after CPPs stereotaxic injection).

### 1-Methyl-4-Phenyl-1,2,3,6-Tetrahydropyridine (MPTP) Monkey Model

Monkeys were treated with 0.5 mg/kg MPTP (i.v., femoral vein) every 2 weeks for a variable period of 2–4 months to reach different degrees of nigro-striatal lesion and parkinsonism (Blesa et al., 2010, 2012) determined by the total number of doses and variable individual susceptibility to MPTP when using a slow intoxication protocol. In this study, eight animals were used as control and 17 monkeys were treated with MPTP. Motor deficit was assessed weekly by Kurlan motor scale which ranges from 0 to 29 (Kurlan et al., 1991). 17 monkeys developed parkinsonian features with the first 1–2 MPTP injections. Five monkeys did not receive any further MPTP injection and exhibited progressive improvement until motor behavior had normalized (score 0–1) and are labeled as recovered monkey (Mounayar et al., 2007). The remaining 12 monkeys continued to receive MPTP as explained above to achieve stable moderate parkinsonism (*n* = 5; Kurlan’s score 8–15) and severe parkinsonism (*n* = 7; Kurlan’s score > 16). Each animal remained stable for at least 1 month in the corresponding motor state before initiating treatment (Blesa et al., 2012).

### L-DOPA-Induced Dyskinesias and TAT2A Treatment in Monkeys

Parkinsonian monkeys with severe symptoms were chronically treated with L-DOPA to induce dyskinesia. L-DOPA (50–55 mg/kg, i.p.) was injected daily until a stable dyskinetic behavior was achieved (6 months). The severity of dyskinesia was scored daily blindly using a scale which is used in the clinical practice. This rating system assesses both severity (0–3) and duration (0–3) of the dyskinetic movements in different body parts including the four limbs, the trunk and the face (Luquin et al., 1992). Animals were sacrificed after 3 months of chronic treatment with L-DOPA for brain tissue analysis. The brain was removed and fresh frozen. Tissue from head of caudate, anterior and posterior putamen was collected for the subcellular fractionation studies.

Three MPTP parkinsonian monkeys previously made dyskinetic with L-DOPA as explained above were used to test the potential antidyskinetic effect of TAT2A. Three TAT2A doses of 3, 4.5 and 6 nmol/g were given intraperitoneally 30 min before L-DOPA administration in different study days.

One monkey received the 3 nmol/g dose twice with no effect whatsoever. Thus, TAT2A dose was increased to 4.5 nmol/g and 6 nmol/g in the three monkeys. Dyskinesia score was measured as described above. A 1-week period in-between peptide injections was taken as washout. L-DOPA treatment was continued on a daily basis.

### Post-Mortem Tissue from Human Subjects

Post-mortem brain material (putamen) from PD patients (*n* = 16; see Table 1) and age-matched control subjects (*n* = 16) were used in this study. Control brains were obtained from individuals without neurologic or psychiatric disorders. Patients with PD displayed the characteristics of the disease including akinesia, rigidity and/or resting tremor. All patients with PD were treated with L-DOPA and/or dopaminergic agonists and/or anti-cholinergic agents and displayed or had displayed dyskinesia (see Table 1). The clinical diagnosis was confirmed by neuropathological examination. Mean post-mortem delay and age at death did not differ between control subjects and patients with PD. At autopsy the brain was removed and hemi-sected: one hemisphere was formalin fixed for neuropathological confirmation of the diagnosis and the other half was dissected and flash frozen. All brains showed neuronal loss in the substantia nigra and Lewy bodies detected by hematoxylin-eosin staining. The putamen was dissected out from the striatum, frozen and reduced to power on dry ice. Frozen tissue was pooled from the whole structure from each patient. Then 10 to 15 mg of putamen were prepared for each subject and used for biochemical measurements.

**Table 1:** **Table summarizing the clinical data of patients from which brain samples were obtained**.

PARK N°	Age (years)	Post-mortem delay (hours)	Sex	Disease duration (years)	Treatment
1	69	24	M	9	Madopar (benserazide hydrochloride/levodopa)
2	66	<26	M	13	Madopar (benserazide hydrochloride/levodopa)
3	59	<24	M	25	Madopar (benserazide hydrochloride/levodopa)
4	82	15	M	6	Madopar (benserazide hydrochloride/levodopa), Parlodel (bromocriptine)
5	57	15	F	15	Madopar (benserazide hydrochloride/levodopa)
6	67	11	M	14	Madopar (benserazide hydrochloride/levodopa), Artane (Trihexyphenidyl)
7	75	8	M	7	Madopar (benserazide hydrochloride/levodopa)
8	77	3	M	15	Madopar (benserazide hydrochloride/levodopa)
9	83	11	F	2	Madopar (benserazide hydrochloride/levodopa), Artane (Trihexyphenidyl)
10	84	10	M	20	Madopar (benserazide hydrochloride/levodopa), Artane (Trihexyphenidyl)
11	69	12	M	16	Madopar (benserazide hydrochloride/levodopa), Artane (Trihexyphenidyl),
12	68	<24	M	2	Madopar (benserazide hydrochloride/levodopa)
13	79	15	F	Unknown	Unknown
14	76	12	M	35	Madopar (benserazide hydrochloride/levodopa), Artane (Trihexyphenidyl)
15	77	<24	M	10	Madopar (benserazide hydrochloride/levodopa)
16	84	37	F	4	Madopar (benserazide hydrochloride/levodopa)

### Subcellular Fractionation

Subcellular fractionation was performed as previously described (Gardoni et al., 2006) with only few modifications. Briefly, striata were homogenized with a hand-held Teflon-glass homogenizer in ice-cold buffer (pH 7.4) containing 0.32 M sucrose, 1 mM Hepes, 1 mM MgCl2, 1 mM NaHCO3 and 0.1 phenylmethanesulfonylfluoride (PMSF) in the presence of Complete Protease Inhbitor Cocktail Tablets (Roche Diagnostics, Basel, Switzerland) and phosSTOP Phosphatase Inhibitor Cocktail Tablets (Roche Diagnostics, Basel, Switzerland). The contralateral striatum of fully 6-OHDA lesioned rats was used as control. An aliquot of the homogenate was stored at −20°C, while the rest of the sample was centrifuged at 800 g for 5 min to remove nuclear contamination and white matter. The resulting supernatant was spun at 13,000 g for 15 min at 4°C to obtain a crude membrane fraction (P2 fraction) as a pellet which was resuspended with a glass-glass homogenizer in 1 mM Hepes supplemented with Complete Cocktail. An aliquot of the P2 fraction was stored at −20°C for co-immunoprecipitation assays, whereas the rest of the sample was centrifuged at 100,000 g for 1 h at 4°C. The new pellet was resuspended in 75 mM KCl and 1% Triton X-100 and spun at 100,000 g for 1 h at 4°C. The final pellet was homogenized in a glass-glass potter in 20 mM Hepes supplemented with Complete tablets and then stored at −80°C. This fraction was referred to as Triton X-100-insoluble fraction (TIF). We isolated the TIF instead of the classical PSD because the amount of the starting material was often limited. The protein composition of the TIF was carefully tested for the absence of presynaptic markers (Gardoni et al., 2006). Similar TIF yields were obtained from striata of all experimental groups.

### Co-Immunoprecipitation Assay

P2 fraction (50 μg) from the striatum of parkinsonian, dyskinetic and control rats were incubated in radio-immunoprecipitation assay (RIA) buffer [200 mM NaCl, 10 mM ethylenediaminetetraacetic acid (EDTA), 10 mM Na_2_HPO_4_, 0.5% Nonidet P-40] supplemented with 0.1% sodium dodecyl sulfate (SDS) and anti-PSD-95, anti-GluN2A or anti-GluN2B antibodies. Samples were incubated overnight at 4°C on a wheel. Protein A/G-agarose beads (Santa Cruz, Dallas, TX, USA) were added and incubation was continued for 2 h at room temperature (RT) on the wheel. Beads were collected and washed four times in RIA buffer containing 0.1% SDS. Laemmli sample buffer was then added to the beads and the mixture was boiled for 10 min. Beads were pelleted by centrifugation and the supernatant was loaded onto an acrylamide/bisacrylamide gel for SDS-PAGE.

### Western Blot (WB)

Protein samples were separated onto 7–9% acrylamide/bisacrylamide gel, transferred to a nitrocellulose membrane and probed with the appropriate primary and HRP-conjugated secondary antibodies. Labeling was visualized by Chemidoc and ImageLab software (Biorad). Western blot (WB) quantification was performed using ImageLab software.

### Free-Floating Immunohistochemistry

5(6)-FAM-conjugated TAT2A peptide was administrated to adult male non-human primates (i.p.) 30 min before sacrifice. Control monkeys were injected with the vehicle. The brain was dissected and post-fixed in 4% paraformaldehyde (PFA) in 0.1 M tris buffered saline (TBS) followed by sequential sinking in 20% and 30% sucrose in TBS. The tissue was then sectioned using a microtome and 40 μm-thick slices were stored in a cryoprotective solution at −20°C until use. Antigens were retrieved with 10 mM sodium citrate pH 6. Tissue sections were permeabilized in TBS containing 0.4% Triton X-100 (0.4% T-TBS) for 30 min at 4°C. After blocking in 5% Normal Goat Serum (NGS, #G9023, Sigma-Aldrich) in 0.1% T-TBS for 2 h at RT, slices were incubated with anti-HIV1 TAT and anti-microtubule associated protein 2 (MAP2) antibodies in 3% NGS-0.1% T-TBS overnight at 4°C. Tissue sections were then incubated with the appropriate AlexaFluor secondary antibodies in 3% NGS-TBS for 2 h at RT. Nuclei were stained with the fluorescent dye 4′,6-diamidino-2-phenylindole (DAPI, Life Technologies, Carlsbad, CA, USA). Slices were finally mounted with Fluoromount mounting medium (Sigma-Aldrich) onto glass slides. Labeling was visualized with LSM510 Meta system confocal microscope (Zeiss, Oberkochen, Germany) and Aim 4.2 software (Zeiss).

### Statistics

Data were analyzed using GraphPad Prism version 6 (GraphPad Software, La Jolla, CA, USA). Data followed a normal distribution and the significance of the differences was analyzed by unpaired two-tailed Student’s *t*-test/one-way or two-way ANOVA followed by Bonferroni or Tukey *post hoc* tests as appropriate. Details of the statistical analysis applied in this work and the *p* values are given in the “Results” Section and/or in the Figure legends. Data are presented as mean ± SEM.

## Results

### Chronic L-DOPA Treatment Increases GluN2A/GluN2B Subunit Ratio at Striatal Synapses in Animals Showing a Dyskinetic Motor Behavior

**Figure 1:**
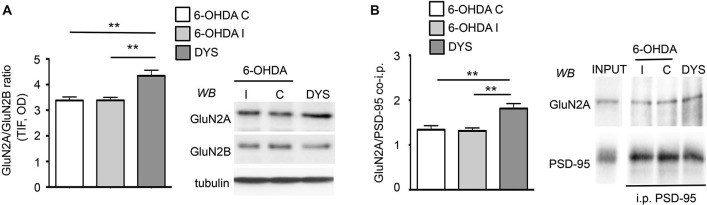
**Chronic L-DOPA treatment increases GluN2A/GluN2B subunit localization at synaptic sites in 6-OHDA rats displaying dyskinetic behavior. (A)** WB for GluN2A, GluN2B and tubulin of TIF samples from the ipsilateral (6-OHDA I) and the contralateral (6-OHDA C) striata of 6-OHDA-lesioned rats and the ipsilateral striatum of L-DOPA-treated (6 mg/kg/die) dyskinetic animals (DYS). GluN2A/GluN2B ratio is increased in dyskinetic rats (**p < 0.01). **(B)** Co-immunoprecipitation of GluN2A and PSD-95 in P2 fractions from the ipsilateral (6-OHDA I) and the contralateral (6-OHDA C) striata of 6-OHDA-lesioned rats and the ipsilateral striatum of L-DOPA-treated (6 mg/kg/die) dyskinetic rats (DYS). The interaction between GluN2A and PSD-95 is augmented in the crude membrane fraction of dyskinetic animals (**p < 0.01).

Levels of NMDAR regulatory subunits were analyzed by WB in homogenate and postsynaptic fraction (Triton-insoluble fraction, TIF) from the ipsilateral (6-OHDA I) and contralateral (6-OHDA C) striata of fully 6-OHDA-lesioned rats and the ipsilateral striatum of L-DOPA-treated (6 mg/kg per day) dyskinetic rats (DYS; Figure 1A). Similar TIF protein yields were obtained from all groups, and the same amount of proteins was used for SDS-PAGE. As previously reported (Gardoni et al., 2006), no differences in the expression level of any of the tested NMDAR subunits were observed in the homogenate from the three experimental groups (data not shown). On the contrary, chronic L-DOPA administration determined a significant increase in the synaptic ratio of GluN2A/GluN2B subunits compared to controls and 6-OHDA-lesioned rats (Figure 1A; ***p < 0.001 one-way ANOVA; Bonferroni *post hoc* test, DYS vs. 6-OHDA I: **p < 0.01, DYS vs. 6-OHDA C: **p < 0.01). These results are in agreement with what was previously described in dyskinetic rats exposed to a higher (20 mg/kg per day) dose of L-DOPA (Gardoni et al., 2006). We then tested whether the observed alterations in GluN2A/GluN2B synaptic abundance correlated with changes in the receptor interaction with members of the membrane-associated guanylate kinases (MAGUK) family such as the scaffolding protein PSD-95. Dyskinetic rats showed an increased binding between GluN2A and PSD-95, thus favoring the anchoring of such NMDAR subtype at the synaptic compartment (Figure 1B; **p < 0.01 one-way ANOVA; Bonferroni *post hoc* test, DYS vs. 6-OHDA I: **p < 0.01, DYS vs. 6-OHDA C: **p < 0.01).

**Figure 2:**
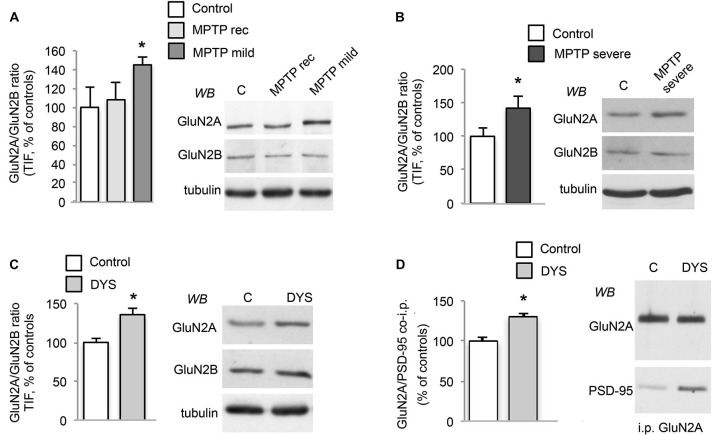
**DA denervation and chronic L-DOPA treatment increases GluN2A/GluN2B subunit ratio at synaptic sites in the MPTP-monkey model. (A)** WB for GluN2A, GluN2B and tubulin of TIF samples from the striatum of control (C), MPTP-treated monkeys with mild motor alterations (MPTP mild; *p < 0.05) and mild parkinsonian monkeys which had fully recovered by interruption of MPTP treatment (MPTP rec). **(B)** WB for GluN2A, GluN2B and Tubulin of TIF samples from the striatum of control (C) and MPTP-treated monkeys with severe motor alterations (MPTP severe; *p < 0.05). **(C)** WB for GluN2A, GluN2B and Tubulin of TIF samples from the striatum of control (C) and dyskinetic MPTP-treated monkeys (DYS; *p < 0.05). **(D)** GluN2A subunit was immunoprecipitated and its binding to PSD-95 was analyzed by WB in the striatum of control (C) and dyskinetic MPTP-treated monkeys (DYS). GluN2A and PSD-95 binding was significantly increased (*p < 0.05).

**Figure 3:**
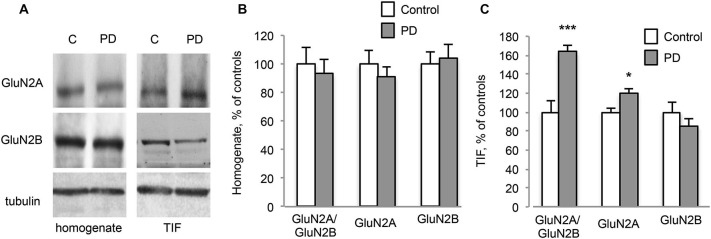
**Chronic L-DOPA treatment increases GluN2A/GluN2B subunit ratio at synaptic sites in dyskinetic Parkinson’s disease (PD) patients. (A)** WB for GluN2A, GluN2B and Tubulin of homogenates and TIF samples from the striatum of control (C) and dyskinetic PD patients (PD). **(B,C)** Quantification of GluN2A and GluN2B subunits in homogenates **(B)** and TIF fractions **(C)** from dyskinetic PD patients and control subjects. The analysis showed a statistically significant increase in GluN2A synaptic localization and the subsequent alteration of GluN2A/GluN2B subunit ratio at synapses of dyskinetic PD patients (*p < 0.05; ***p < 0.001). No changes were found in homogenates.

The 1-methyl-4-phenyl-1,2,3,6-tetrahydropyridine (MPTP)-treated macaque is still considered the gold standard animal model for PD as it shares several features of the human disease (Bezard and Przedborski, 2011; Blesa et al., 2012; Porras et al., 2012). Accordingly, to strengthen the relevance of the data from the rodent experimental model, we decided to expand our analysis on NMDAR subunit composition to MPTP-treated monkeys (Figure 2) and post-mortem brain tissue from dyskinetic PD patients as a key step for target validation (Figure 3). Protein levels of NMDAR subunits were evaluated by WB in postsynaptic fractions from the striatum of control and MPTP-treated monkeys with mild or severe motor alterations. GluN2A/GluN2B ratio was significantly increased at synapses from both MPTP-mild (Figure 2A) and severe (Figure 2B) parkinsonian monkeys (MPTP-mild or MPTP-severe vs. control animals: *p < 0.05, two-tailed unpaired Student’s *t*-test). In contrast, NMDAR subunit levels were normal in moderately lesioned MPTP monkeys (Figure 2A; MPTP rec group vs. control animals: *p* > 0.05, two-tailed unpaired Student’s *t*-test).

In addition, abnormal GluN2A/GluN2B ratio at striatal synapses was also detected in L-DOPA-treated MPTP-monkeys displaying a dyskinetic motor behavior (Figure 2C; DYS vs. controls: *p < 0.05 two-tail unpaired Student’s *t*-test). No changes in NMDAR subunit expression in the homogenate from MPTP-mild, MPTP-severe or L-DOPA-treated dyskinetic animals were found when compared to control monkeys (data not shown). Finally, when we checked for the interaction with the scaffolding protein PSD-95 in the dyskinetic macaques, we observed an increased binding of GluN2A to PSD-95 (Figure 2D; GluN2A/PSD-95 DYS vs. controls: *p < 0.05), which was not replicated with GluN2B (data not shown). Altogether, these results indicate that NMDAR composition is reorganized not only in the rat model but also in this highly validated primate model of PD and LIDs.

Furthermore, post-mortem material from the putamen of dyskinetic PD patients (see Table 1) and age-matched control subjects (C) was analyzed by WB (Figure 3A). No changes in NMDAR GluN2A and GluN2B subunit levels were detected in the homogenates (Figures 3A,B; PD vs. C: *p* > 0.05, two-tailed unpaired Student’s *t*-test). However, PD patients were characterized by a significant increase in the synaptic localization of GluN2A but not GluN2B (Figures 3A,C, TIF fraction; GluN2A PD vs. C: *p < 0.05, GluN2B PD vs. C: *p* > 0.05, two-tailed unpaired Student’s *t*-test). Overall, these changes led to a significant increase in GluN2A/GluN2B subunit ratio at synaptic sites in PD patients compared to control subjects (Figures 3A,C, TIF fraction; GluN2A/GluN2B PD vs. C: ***p < 0.001, two-tailed unpaired Student’s *t*-test).

Altogether, our data highlight molecular modifications of NMDAR composition in two different models of experimental parkinsonism and LIDs, and in the human pathology. Moreover, changes in the interaction between the receptor subunits and members of the MAGUK family are present in 6-OHDA dyskinetic rats and MPTP dyskinetic monkeys.

### TAT2A Peptide Ameliorates the Severity of Established Dyskinesias through the Dissociation of GluN2A Subunit from PSD-95

**Figure 4:**
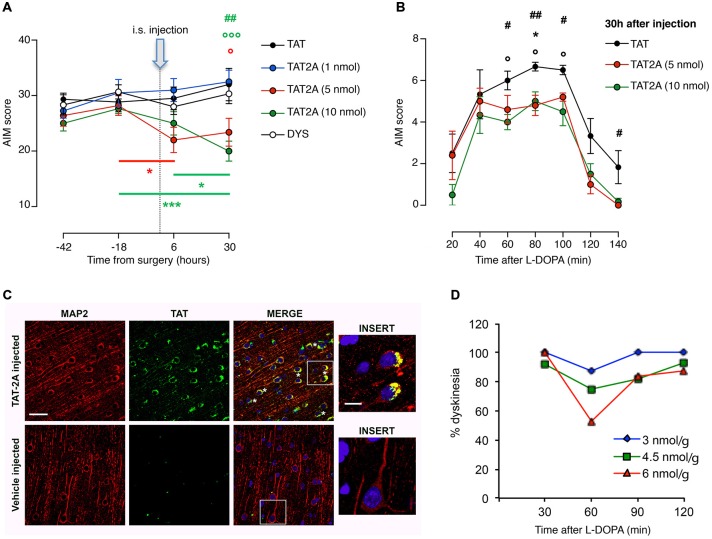
**TAT2A peptide ameliorates the severity of established dyskinesia. (A)** Decrease in the dyskinesia score of 6-OHDA rats treated with L-DOPA after 6 h and 30 h from the intrastriatal injection of TAT2A (^*,°^*p* < 0.05, ^##^*p* < 0.01, ^***, °°°^*p* < 0.001). **(B)** Reduction of AIMs induction in TAT2A 5 or 10 nmol injected animals in the session test 30 h after CPP injection (^*,°,#^*p* < 0.05, ^##^*p* < 0.01). **(C)** Immunohistochemistry for TAT sequence (green) and MAP2 (as neuronal marker, red) from the brain of monkeys treated with a 5(6)-FAM-conjugated TAT2A peptide or the vehicle. Nuclei were labeled with DAPI (blue). Scale bar: 40 μm; Insert scale bar: 10 μm. **(D)** Reduction of the dyskinesia score in MPTP-monkeys after i.p. TAT2A treatment 30 min before L-DOPA injection. The graph shows dyskinesia scores (Y-axis) over the time course of levodopa (X-axis) at the three doses of TAT2A (3 nmol/g; 4.5 nmol/g; 6 nmol/g) in the 3 MPTP monkeys expressed as percentage of the dyskinesia score before TAT2A treatment.

To rebalance synaptic NMDAR composition, we focused on the mechanisms regulating GluN2A localization at the synaptic compartment. In particular, we took advantage of the CPP approach to disrupt GluN2A binding to scaffolding proteins of the MAGUK family. We have previously shown that a chronic concomitant treatment with L-DOPA and a CPP targeting GluN2A/PSD-95 interaction (TAT2A) was able to reduce the number of 6-OHDA-lesioned rats developing dyskinetic movements (Gardoni et al., 2012). However, from a clinical point of view, to determine the anti-dyskinetic potential of a compound, it is necessary to evaluate its ability to reduce the severity of already established LIDs. Therefore, we evaluated whether administration of TAT2A to dyskinetic rats after chronic treatment with L-DOPA could have beneficial effects on their behavior. TAT2A or TAT control peptides were stereotaxically injected in the ipsilateral striatum of 6-OHDA-lesioned rats chronically treated with L-DOPA and displaying dyskinetic motor behavior (AIMs score ≥ 25; see “Materials and Methods” Section). CPPs were injected 6 h before the daily L-DOPA administration and the evaluation of AIMs was carried out from 20 to 140 min after L-DOPA (Figures 4A,B). 5 and 10 nmol TAT2A were able to induce a significant reduction of the AIMs score (Figure 4A; two-way ANOVA *F*_(8,44)_ = 3.076, *p* < 0.01; Tukey *post hoc* test, TAT2A 5 nmol: −18 vs. 6 h, *p < 0.05; TAT2A 10 nmol: −18 vs. 30 h, ***p < 0.001, 6 vs. 30 h, *p < 0.05. 30 h: TAT vs. TAT2A 5 nmol, °*p* < 0.05, TAT vs. TAT2A 10 nmol, °°°*p* < 0.001, TAT2A 10 nmol vs. DYS, ##*p* < 0.01). Moreover, the time course of AIMs development showed that both TAT2A peptide dosages significantly decreased AIMs induction, measured during the single last observation session (Figure 4B; 30 h: One-way ANOVA, Tukey *post hoc* test: 20 min: *p* > 0.05; 40 min: *p* > 0.05; 60 min: #*p* < 0.05, TAT vs. TAT2A 10 nmol, °*p* < 0.05; 80 min: ##*p* < 0.01, TAT vs. TAT2A 10 nmol, °*p* < 0.05, TAT vs. TAT2A 5 nmol *p < 0.05; 100 min: #*p* < 0.05, TAT vs. TAT2A 10 nmol, °*p* < 0.05; 120 min: *p* > 0.05; 140 min: #*p* < 0.05). No effect was observed following injection of 1 nmol TAT2A in agreement with previous data (Gardoni et al., 2012). No difference was observed in rats treated with TAT control peptide compared to untreated dyskinetic rats, thus demonstrating the absence of any effect induced by the surgery procedure or by the TAT moiety (Figure 4A).

To assess the potential therapeutic effect on the non-human primate model, we tested TAT2A peptide on a limited number of dyskinetic monkeys (*n* = 3). Before the evaluation of the effect of TAT2A on the motor behavior, we first verified the ability of this CPP to cross the blood brain barrier, reach the brain and enter neurons. One monkey was injected (i.p.) with a labeled TAT2A peptide while another one received the vehicle 30 min before their sacrifice. The prefrontal cortex was fixed, sliced and immunostained with an antibody against the TAT sequence (green) and MAP2 (as neuronal marker, red; Figure 4C). While no positive staining for TAT was detectable in the control macaque (vehicle-injected), immunolabeling demonstrated that TAT2A is able to reach the brain of non-human primates and the peptide was clearly found in neuronal cells located in the cortex (Figure 4C, insert). Monkeys that received L-DOPA (50–55 mg/kg i.p.) for 6 months and exhibited a persistent dyskinetic behavior were treated systemically with TAT2A peptide at the doses of 3, 4.5 and 6 nmol/g 30 min before the daily L-DOPA administration. Comparably to the TAT2A treatment in rats, the higher dose used in this study (6 nmol/g) was able to reduce the dyskinetic behavior with the highest effect 60 min after L-DOPA injection. At this dose we observed an average reduction in the dyskinesia score of 47.15% (*n* = 2, 2 days, Figure 4D). A 25% reduction was found at 60 min with the 4.5 nmol/g dose (*n* = 3, 2 days), while 12.5% reduction was evident with 3 nmol/g TAT2A (*n* = 1, 2 days; Figure 4D). The anti-parkinsonian effect of L-DOPA was maintained in the co-treatment with TAT2A (data not shown).

## Discussion

Altered subunit composition of striatal NMDARs have been found to be involved in the pathophysiology of LIDs (Gardoni et al., 2006, 2012; Calabresi et al., 2010; Mellone and Gardoni, 2013). However, a proof of concept of this hypothesis in well-established rodent and primate models of LIDs as well as a validation in human post-mortem tissue was not complete. Our results demonstrate that a single intrastriatal injection of a peptide (TAT2A) capable of reducing synaptic accumulation of GluN2A-containing NMDARs leads to a dose-dependent decrease of AIMs in L-DOPA-treated dyskinetic rats. Notably, systemic administration of TAT2A peptide to MPTP-treated monkey with established LIDs determined a dose-dependent reduction of dyskinetic behavior comparable to what was observed in the rat 6-OHDA model. These results indicate a cross-species validation of the efficacy of this therapeutic approach.

So far NMDAR subunit expression in L-DOPA-treated MPTP-lesioned non-human primates (Calon et al., 2002; Hallett et al., 2005; Morin et al., 2013) and PD patients (Holemans et al., 1991; Weihmuller et al., 1992; Calon et al., 2003) has not been extensively investigated, and the few studies available have been mainly carried out using a receptor binding approach with radiolabeled ligands. Among these reports, only a previous study from Hallett et al. (2005) analyzed NMDARs subcellular distribution and they found that dopamine depletion and subsequent replacement with L-DOPA in MPTP-lesioned macaques induces significant alterations in the abundance of striatal NMDAR subunits. In particular, following chronic treatment with L-DOPA, they observed a striking increase in the abundance of GluN2A subunit in the synaptic membrane fraction (Hallett et al., 2005). The significant increase in GluN2A/GluN2B subunit ratio that we found in both parkinsonian and L-DOPA-treated dyskinetic monkeys is consistent with these previous observations (Hallett et al., 2005). Moreover, we observed a similar increase of synaptic GluN2A/GluN2B ratio in dyskinetic PD patients which highlights the relevance of therapeutically targeting GluN2A clustering at synapses. Conversely, it is challenging to compare our results with studies employing a ligand binding approach to detect levels of NMDAR subunits. Previous studies on MPTP-treated non-human primates (Calon et al., 2002; Morin et al., 2013) did not observe significant changes in the binding of ligands to the GluN2A subunit. Considering that these studies were not performed in a fractionated tissue samples, it is reasonable to compare them with our western blotting performed in a total cell homogenate where we did not find any alteration of GluN2A or GluN2B subunits in parkinsonian as well as L-DOPA-treated dyskinetic macaques. Similarly, no alterations in GluN2A/GluN2B ratio from total striatal homogenates were detected in monkeys, and notably in patients’ post-mortem tissue. These results further support the idea that an abnormal synaptic localization of specific subtypes of NMDARs rather than their altered expression level represents the main event taking place in MSNs excitatory synapses after L-DOPA administration. Altogether, the above considerations support the applicability of CPP interfering with GluN2A subunit clustering at the excitatory synapse as therapeutic agent instead of the classical receptor antagonist approach, which inhibits the activity of surface NMDARs at both synaptic and extrasynaptic sites. Accordingly, the previous failure to prevent the development of LIDs in MPTP-treated primates using the GluN2A antagonist MDL100,453 (Blanchet et al., 1999) can be explained both by the low degree of selectivity for GluN2A over GluN2B of the antagonist as well as by the lack of specificity towards synaptic NMDARs.

We previously reported that the concomitant chronic administration of L-DOPA and TAT2A was able to prevent the onset of LIDs in the rat model (Gardoni et al., 2012). Here we extend these earlier results, showing the efficacy of TAT2A treatment in reducing LIDs also in animals with established and consolidated dyskinesia. Notably, the absence of any effect observed with single intrastriatal injection of 1 nmol TAT2A is in agreement with the previous observation of a lack of anti-dyskinetic activity following repeated injection of 0.5 nmol TAT2A (Gardoni et al., 2012) and indicates an intrastriatal injection of 5–10 nmol as the most appropriate dosage. Interestingly, a previous study from our group also showed that TAT2A rescued PD motor symptoms and restored altered striatal plasticity in 6-OHDA rats with a partial nigrostriatal lesion (Paillé et al., 2010).

In the last decade, the clinical administration of CPPs has been debated because of pharmacokinetic and economic reasons. However, promising results from recent studies have been supporting their use in preclinical and clinical stages, clearly highlighting their therapeutic relevance (Bach et al., 2011; Hill et al., 2012; Mellone and Gardoni, 2013). An analogous peptide (Tat-GluN2B9c) targeting GluN2B-containing NMDARs interaction with PSD-95, which differs only by two amino acids with the TAT2A peptide used in our study, was recently tested in a phase 2 randomized double-blinded placebo-controlled clinical trial for stroke (Hill et al., 2012). Hill and co-authors demonstrated that Tat-GluN2B9c can be safely administered to patients, strengthening the importance of interference peptides in central nervous system disorders paving the way for their future therapeutic application (Hill et al., 2012). In addition, the production of small and potent plasma-stable peptidomimetic compounds deriving from CPPs (Bach et al., 2011) represents the possibility for a further improvement and a more favorable clinical applicability of this therapeutic intervention.

Our results demonstrate that altered GluN2A/GluN2B ratio at striatal synapses represents a synaptic trait of LIDs in different experimental models as well as in PD patients and indicate that reduction of GluN2A synaptic abundance can represent a novel strategy to counteract LIDs.

## Author Contributions

MM, JS, BP, MDL and FG designed research; MM, JS, EZ and AL performed all research in the rat model and biochemical analysis on monkey and human samples; LFH and EI performed the monkey behavioral study; JO supervised and designed the monkey study; MM, JS, LFH and FG analyzed data; MM, JS, MDL and FG wrote the manuscript; LFH, BP and PC assisted with the editing of the manuscript; AP and ECH provided the postmortem tissue from PD patients, and assisted with the editing and proofreading of the manuscript.

## Conflict of Interest Statement

The authors declare that the research was conducted in the absence of any commercial or financial relationships that could be construed as a potential conflict of interest.
